# A Question of Contact Isolation

**Published:** 1920-06-05

**Authors:** 


### A SMALL-POX POLICY.

### A Question of Contact Isolation.

SHOULD small-pox contacts be isolated? This point is raised by the Camberwell Borough Council, which has been asked by the Ministry of Health for its observations on a communication which the London County Council sent to the Ministry with reference to the question of accommodation in Camberwell for the isolation of small-pox contacts.

In its letter to the Health Ministry the L.C.C. refers to the opinion held by Camberwell that the visitation of contacts by the sanitary inspector is to be preferred to the isolation of contacts in a suitable building, and proceeds to state that it is advised that the method of successfully preventing the spread of small-pox is medical inspection, with a view to the detection of the disease in its earliest stages, and that for this purpose contacts should in certain cases be kept under close medical supervision up to sixteen or eighteen days from the possible date of exposure to risk of infection. The L.C.C. considers that special accommodation should be available in order that the thorough disinfection of infected premises and articles should be carried out, and the contacts kept under the strict personal observation of the medical officer of health. The L.C.C. also refers to Camberwell's objection that the shelter at Peckham Park Road depôt is unsuitable for use in the manner suggested, and states that for many years past it has been the practice in many London boroughs at times of small-pox prevalence to use the shelters which Borough Councils are under statutory obligation to provide for accommodating small-pox contacts in case of need. The L.C.C. adds that if Camberwell regards the existing premises as unsuitable for this purpose, it should endeavour to obtain alternative reserve accommodation, or, failing that, adapt the existing shelter.

The Borough Council has now had the subjoined statement by Dr. F. Stevens, its medical officer of health, and suggests that this should be sent to the Ministry of Health:

"In my report to the Borough Council of October 8, 1918, I advised that daily visits should be made by the inspectors to houses where cases had occurred, and any suspicious symptoms should at once be reported to me, hence the statement in the last letter from the London County Council that the visitation of contacts is to be preferred to their isolation might, of course unintentionally, convey a wrong impression that visitation by the inspector is all that is necessary. Granting, however, that the theories of the London County Council are correct, we are met by the difficulty of obtaining suitable accommodation. Even if we could find a house, there would have to be provision for more than one family, so as to minimise the risk of shutting up of separate families in a strange house with little to do. However, it is idle to talk about a house in the neighbourhood of the cleansing station, as I believe it is impossible to get even an unsuitable one. It is not necessary to enlarge, therefore, on the domestic difficulties likely to arise in these cases, and on the lack of effective control, which could only be obtained not by liberal bribery with tobacco and other luxuries, but by a practically military discipline. We are therefore driven to the second alternative. It is impossible to consider as suitable for a shelter rooms where the same conditions obtain as regards control, and which are practically in direct communication with the station for the cleansing of school children, and which cannot be shut off from the depôt so as to prevent the mingling with the Council's employees of those persons who are considered to be so suspicious as to call for the personal supervision of the medical officer of health.

"I take it that this Council is every whit as anxious as any other body to take all possible means to prevent the spread of small-pox, but if they are to be held responsible as the local sanitary authority they surely are entitled to use some discretion as to whether the opinions of the London County Council are the only ones that are to be followed. If an authoritative decision is given by the Ministry of Health, on their responsibility, it would be for this Council to take action on the lines laid down, but not before. If the London County Council are so impressed with the necessity for these shelters, it should not be impossible for them to arrange with the Metropolitan Asylums Board for their provision in such districts irrespective of local areas to which any cases they think necessary could be admitted. So far as the presumed necessity for accommodation while thorough disinfection of premises and articles is carried out, there has never been the slightest difficulty in obtaining this."

It will be interesting to note what conclusion is finally drawn upon this matter, for in practice we believe that general opinion of medical officers of health of similar boroughs will support Dr. Stevens. We should be glad to hear their views.

